# The Institutional Library

**Published:** 1918-07-06

**Authors:** 


### THE INSTITUTIONAL LIBRARY.

### Hysterical Disorders of Warfare.

**By LEWIS R. YEALLAND, M.D.** (London: Macmillan and Co., Ltd. 1918. Pp. 252 + xii. Price 6s. net.)

IN a Preface written by Dr. Farquhar Buzzard we are told that the author of this book, having become interested in psychological problems as an outcome of asylum work pursued in Canada, came to London for the special purpose of studying neurology. His arrival was opportune, for the war was beginning to provide numerous and varied examples of paralytic and other disturbances depending not on organic but on functional disease. Here, then, was an admirable opportunity of studying both psychology and neurology, and it is evident that Dr. Yealland seized it with characteristic vigour. He now tells us of his experiences, and describes in detail the methods by which he achieves his therapeutic triumphs. Indeed, his book is mainly a description of the successful treatment of individual cases of such conditions as hysterical mutism, aphonia, stammering, hemiplegia, paraplegia, blindness, fits, blepharospasm, and so on. The interviews between doctor and patient are given *verbatim*, and the "encounters," as Dr. Buzzard names them, have a truly dramatic quality. Indeed, they can hardly fail to remind the reader of the transatlantic police methods that are grouped under the term "the third degree." The patient has to learn that he is in the presence of a master, and that before he escapes he must do what he is told. For the most part it is in the single interview that the triumph is gained, and whatever electrical or other apparatus may be used it is the influence of the man, not that of the machine, which is the dominating factor in the therapeutic success. Of course, it is a large thing to cure these cases at all, but to cure them with promptness, expedition, and certainty is indeed an added gain, and this especially just now, when the victims are so numerous and so helpless. The book is a most interesting and graphic one. But not every physician will be equal to the method, and, indeed, to some the method may be repugnant. To hammer away for four continuous hours at a young man suffering from mutism, the programme including a dark room, electrical shocks, and sustained authority, exhortation and command, is a serious undertaking, and must mean both mental force and physical vigour. Yet success is surely worth all the trouble, for it occurs in cases which hitherto have been the despair of ordinary therapy, the failure being all the more bitter as it is attended with the conviction that the patient has no structural defect and is quite capable of leading a happy and useful life. We congratulate Dr. Yealland on the success of his work, and we are glad that he has so frankly and in such detail described his methods for the benefit of others. Hysterical paralysis and its like are just now much with us, and their prompt cure is a matter of concern, not only to the patients themselves, but also to the Ministry of Pensions and the National Exchequer.

**With the R.A.M.C. in Egypt.** By SERGEANT-MAJOR, R.A.M.C. (London: Cassell and Co., Ltd. 6s. net.)

THIS book will deeply interest all who read it, whether they have served with the R.A.M.C. in the Mediterranean area or whether they have not. The first category will find that the book will remind them of days spent in those glamorous regions of sunshine and rainstorm, of burning sands and choking dust and clinging mud; will recall laborious days and nights, and pleasant gossip of an evening under star-spangled skies when the moon was bright and Venus and Jupiter hung like lamps on opposite horizons. The second will learn easily and delightfully how the work of the R.A.M.C. is done and the conditions under which it is carried out. The book is vivid. Its language is plain. Its descriptions are convincing. First is a slight sketch of work in days gone by from the 'nineties onward, and something of the wars, great then but dwarfed now, we fought to free the Egyptians from tyranny and an always present fear of a fierce irruption from the Southern deserts. Then come descriptions of Gallipoli and the awful times our men endured on the terrible peninsula. Then an account of the war as seen in Egypt and in Sinai and of fighting on the great old road in and out of Egypt. All through, woven in and out or collected as towards the end, are minute descriptions of the actual work and organisation of the Medical Service. It is well all should read and learn what that work is and how it is done. Ignorance must misunderstand. Knowledge must give clear views. Who has read this work will plainly see that the Medical Service cannot be a refuge for weaklings and derelicts. The author is strong on the point. How is a feeble man, too weak to fight, to carry a stretcher and a twelve-stone man on it over rough ground or through deep sand or through heavy mud? How is a man with only the courage of a conscientious objector to endure the strain of standing-by under heavy fire with no chance to hit back? The work of the R.A.M.C. is for the strong and brave, or else the brave and strong who have fallen fighting the foe must suffer more than they need.

Excellent and exciting are the accounts of the duties of the regimental medical officers and the stories illustrating the dangers and difficulties of the advanced posts of the R.A.M.C.

There are excellent pictures in the book that vividly illustrate the text and show a succession of scenes from life in the field. Certainly no one who has any interest in medical work in the Eastern area of operations or who wishes to be instructed therein should fail to get and read this excellent account of it.

**Symptoms and their Interpretation.** By Sir JAMES MACKENZIE, M.D., F.R.S. Third Edition. (London: Shaw and Sons. 1918. Pp. 318 + xx. Price 8s. 6d. net.)

SIR JAMES MACKENZIE always commands a large and interested audience. Hence there is no room for surprise at the arrival of his book in the dignity of a third edition. Apart from the professional position of the author, the subject of the volume has a special measure of attraction, for the recognition and interpretation of symptoms is the daily task with which the practitioner is confronted. Pain or other abnormal sensation for which no physical interpretation can be discovered is a standing problem in diagnosis, and any careful study which endeavours to afford help in this situation cannot fail of a welcome. Such has been the experience of Sir James Mackenzie's book, which both tells us of his own studies and views and also suggests further lines of clinical observation. The doctrine of referred or reflex pain is by no means new, as readers of Hilton's classical Lectures on Rest and Pain will allow; but it is here worked out in much detail in reference to the thoracic and abdominal viscera, and the work is done with great thoroughness. We are far from questioning the clinical value of the enterprise, but it

**The Institutional Library**—(*continued*).

still remains true that conclusions which have a foundation limited purely to a patient's description of his sensations do not stand in a very confident position. The value of personal testimony here, as elsewhere, varies much, and this factor introduces into such descriptions an element of uncertainty. Still, it is probably true that more could be secured in this direction than is commonly attempted, and Sir James Mackenzie, both by precept and example, is a stimulating influence towards this end. By the study of his book the practitioner will be encouraged to observe and to consider certain evidences of disease which in many volumes receive but a scanty measure of attention.

**Burns and their Treatment.** By J. M. H. MacLeod, M.A., M.D., F.R.C.P. (Oxford University Press. 6s. net.)

The dedication of this little book "to our heroic airmen who in spite of crashes and burns still carry on" sufficiently explains the nature of the work and the source from which much of the material has been gathered. The dermatologist, like the surgeon, has learned much from his experiences in the present war, and here we have, in attractive form—print, paper, and illustrations leaving nothing to be desired—the latest word from one who holds a high place in his subject. There is much that will be of special interest to the skin man proper, but the book is also of the greatest service from the general practitioner's point of view, since the author devotes very considerable space to the subject of the general treatment of burns. He rightly says "the treatment of burns has undergone a veritable revolution during the last few years. The old-fashioned methods with greasy applications and occlusive dressings have given way to a more rational and 'open method' of treatment. . . ." The family doctor is constantly being faced with cases of burns of varying severity, and in this book he will find in easily accessible form all that he needs to ensure his patient having the best-known methods of treatment both for the local condition and also for his general comfort—an aspect of the subject which is of special importance in this class of injury, and one which in the older methods of procedure was too largely ignored.

**The Panel Doctor: His Duties and Perplexities.** By T. M. Tibbetts, M.D. (Lond.), D.P.H. (London: John Bale, Sons and Danielsson, Ltd. Second Edition. 2s. 6d. net.)

Whether it is possible for any one man to understand the National Insurance Act in all its bearings and ramifications may well be open to doubt. Certain it is that the vast majority of panel doctors are constantly confronted by points that are not easy of solution. To mention one aspect of the question which naturally possesses a very particular interest for them—remuneration—we doubt very much whether more than a small percentage of the practitioners concerned would be able to elucidate the figures on their quarterly return paper. Doubtless, the usual procedure is that the statement is bolted whole, the receipt signed, and the cheque posted to the bank. There may be a feeling that some of that 9s. has lost its way, but—well, what's the use of worrying about it! There are other points, however, which cannot be dismissed with a shrug of the shoulders, since they concern the patients as well as the doctor. In the little book before us we have an admirable guide for the practitioner in moments of doubt and perplexity. The author covers practically all the ground which the panel doctor is likely to have to traverse; and there is a mine of information which cannot but save the uncertain medico much worry and many mistakes. This applies particularly to the man newly entering upon panel practice. Instead of having to learn at the price of experience, he may, by reading carefully through this book at the outset of his career, avoid the numberless pitfalls which have in so many cases caused loss of time and temper. Dr. Tibbetts has done the panel doctors a real service, and his book should enjoy a very large sale.

**Staying the Plague.** By N. Bishop Harman, M.A., M.B. (Cantab), F.R.C.S. Pp. 120. Sm. 8vo. (London: Methuen and Co. 1917. Price 1s.)

Mr. Harman is the general editor of Messrs. Methuen's Health Series of shilling books, some of which have impressed us by no means favourably, for they have evidently been written, not because the writer had something to say, but because he had to say something. A subject large enough to justify a paragraph has been stretched and stretched to the dimensions of a book, and has cracked in the process. This cannot, however, be said of the booklet under review. Mr. Harman has reserved for himself the most important subject treated of in the Series, and has treated it admirably. He has allotted to some of his colleagues subjects about as important as What to Do when it Rains or Which Shoe to Put on First; but no one can say that the subject of Venereal Diseases is not big enough to justify a book about them for popular use, and for this purpose the dimensions as well as the manner of Mr. Harman's book are just right. To write on this subject a book for the information of the public at large, containing all that the public ought to know and no more than is necessary, to treat the subject thoroughly, and of it with decency, so that it can be commended with propriety to a lad or a girl, is by no means easy, and Mr. Harman has performed the task extremely well. It is easy to imagine the orgy of salacious suggestion and filthy description, the insistence on the necessity of consulting the author professionally, that would occupy a book on the subject written by one of the Viennese school, brought up in the tradition of Krafft-Ebing and Freud, and it is due to Mr. Harman to acknowledge the high level to which he has lifted the treatment of his subject, the fairness with which he has stated the case, neither exaggerating nor understating its gravity, and the excellence of the advice he gives. The book can be thoroughly and warmly commended.

**Selected Poems.** By Robert W. Service. With some Account of his Life and Experiences. (London: T. Fisher Unwin, Ltd. Price 6d.)

Mr. Service, we learn from this pamphlet, began life in a Glasgow bank, which he left in a fit of discontent at the age of twenty-one, when he sailed in the steerage to Vancouver. After various odd jobs and a tramp in Mexico he again entered a bank, and was sent to Yukon at the time of the gold boom there. As a result of his experiences and the influence of Kipling he began to write verse, which seems to have been very popular. On its profits he wandered in the Balkans, Turkey, and Paris, and was writing more verse and some fiction when the war broke out. He joined the Anglo-American Ambulance Corps, and is now, we learn, with the Second French Army at the Front. The selection of the poems here printed shows that, like most of Kipling's disciples, Mr. Service has kept the slang but not the style of his master. The metres lollop along, but have none of Kipling's rhetorical gallop; and the subjects of the poems are such familiar texts as "Playing the Game," "The Call of the Wild," and so forth. It would be difficult to account for the popularity of the poems were not popular poetry the most extraordinary product on the market, like margarine, cheap, guaranteed pure, and singularly tasteless.

(*Continued on p.* 304.)

### The Institutional Library (*continued from p.* 306).

**The Road to a Healthy Old Age.** By T. BODLEY SCOTT, M.R.C.S. (Eng.), L.R.C.P. (Ed.). London: T. Fisher Unwin. 4s. 6d. net.)

SOME books, perhaps the majority, leave upon the reader's mind a definite impression, which may be favourable or the reverse; but at any rate he knows what he thinks of them, and can express his opinion without undue trouble. On the other hand, one occasionally comes across books that elude one. They leave but a vague, nebulous feeling of dissatisfaction which baffles analysis. Such is the volume before us. We have read it through twice with some care, but still it fails to bite. This is largely due to the first chapter, and were this different we should have avoided the unfavourable first impression. Unfortunately, first impressions, be they favourable, unfavourable, emphatic, or vague, are not easily overcome. Our advice would be that the reader who takes this book in hand should read the first chapter last, otherwise the fear expressed by the author, that "some of my readers may get no further than this first chapter," may prove to be not groundless. It is lacking in cohesion, and some of the attempts at theological disquisition are deplorable. It might well be re-written and amalgamated with Chapter III. in any subsequent edition. This said, we can heartily recommend the other chapters, which deal with the subject arteriosclerosis, upon which the book was really written. The best chapter, in our judgment, is that on "The Treatment and Prevention of Premature Senility," in which the author discusses at length and with considerable ability both the treatment of the pre-sclerotic and the definitely atheromatous stages of arterio-sclerosis by means of the various gland extracts, and the means whereby the condition may be delayed, if not altogether avoided. The book is written both for medical and lay readers, and this is not an easy thing to do successfully. There are passages which will be above the heads of most laymen, but on the whole they may read it with interest and may carry into practice with benefit the information it gives. The chapters on "The Value of Foods" and "Chronic Bronchitis and Bronchial Asthma" will also repay perusal. We are sorry to be unable enthusiastically to speak of the book as a whole, because there is so much that is really valuable. In the matter of Chapter I. Mr. Scott really must try again.

**Clinical Disorders of the Heart Beat.** By THOMAS LEWIS, M.D., D.Sc. Fourth Edition. (London: Shaw and Sons. 1918. Pp. 120 + xii. Price 6s. net.)

THE study of disorders and diseases of the heart has of recent years been brought under the sway of graphic methods, and undoubtedly with very great advantage. There is just the danger that this development may seem to the practitioner to be wholly detached from practical ends, and he may thus rule it out of the chapter of his daily work. The apparatus required is somewhat elaborate and needs practice in its application; the records obtained demand skill in their interpretation; and, as was to be expected, with new conceptions a new vocabulary has arisen. Hence there is a possibility of the establishment of a gap between the new truths and the ordinary practice of medicine. Such a result would be an unfortunate one alike for practitioners and for patients. Dr. Lewis has written his book with the object of bridging this gap. He endeavours to show that to a very large extent the conclusions established by the graphic method can be reached apart from apparatus and by the ordinary procedures of clinical examination. In doing this he has rendered a considerable service to practical medicine, and the appreciation of his effort is shown by the appearance of his book in a fourth edition.

